# Fast Colorimetric Detection of H_2_O_2_ and Glucose: A Way Based on Magnetic Nanoparticles Composed of Fe_3_(PO_4_)_2_·8H_2_O Isolated from *Burkholderia cepacia* CG-1

**DOI:** 10.3390/ijms252312518

**Published:** 2024-11-21

**Authors:** Mingyu Jia, Jueyu Wang, Yuxuan Liu, Daizong Cui, Min Zhao

**Affiliations:** College of Life Science, Northeast Forestry University, Harbin 150040, China; 18724626298@163.com (M.J.); 18804503512@163.com (J.W.); liu1959146010@163.com (Y.L.)

**Keywords:** magnetic nanoparticles, Fe_3_(PO_4_)_2_·8H_2_O, peroxidase-like activity, colorimetric detection

## Abstract

In this study, Fe_3_(PO_4_)_2_·8H_2_O magnetic nanoparticles (MNPs) were successfully extracted from the strain *Burkholderia cepacia* CG-1. We subsequently characterized their composition, structure, and morphology, revealing that these nanoparticles consisted of Fe_3_(PO_4_)_2_·8H_2_O with an average diameter of 66.87 ± 0.56 nm. Our measurements indicated magnetic parameters of 151 Oe for coercivity, 2 emu/g for saturation remanence, and 16 emu/g for saturation magnetization. Our findings confirmed that these magnetic nanoparticles exhibited intrinsic peroxidase-like activity, catalyzing the oxidation of 3,3,5,5-tetramethylbenzidine (TMB) in the presence of H_2_O_2_. Compared to horseradish peroxidase (HRP), the peroxidase mimic demonstrated greater stability under various physicochemical conditions. To investigate the mechanisms underlying the peroxidase-like catalysis of Fe_3_(PO_4_)_2_·8H_2_O, we employed fluorescence spectroscopy and electron spin resonance (ESR) analysis. The results revealed that the peroxidase-like activity of Fe_3_(PO_4_)_2_·8H_2_O stemmed from the generation of hydroxyl radicals (·OH). Furthermore, we established a platform for the colorimetric detection of H_2_O_2_ and glucose. Our method was capable of detecting H_2_O_2_ concentrations as low as 1.0 × 10^−3^ mmol/L. Impressively, this sensitive method was successfully applied to determine glucose levels in human serum.

## 1. Introduction

In recent years, nanomaterials have garnered significant attention for their diverse applications, thanks to their unique physical, chemical, photochemical, and biological properties [1]. One notable biological characteristic of nanomaterials is their enzyme-like activity. In 2007, Gao et al. [2] were the first to report that Fe_3_O_4_ magnetic nanoparticles (FMNPs) exhibited intrinsic enzyme mimetic activity, similar to that of natural peroxidases. These nanoparticles were found to catalyze the oxidation of various peroxidase substrates, including TMB and di-azo-aminobenzene (DAB). Subsequently, numerous other inorganic nanomaterials, such as gold nanoparticles, platinum nanoparticles, CuS nanoparticles, and Co_3_O_4_ nanoparticles, have been investigated for their high catalytic efficiency, which is attributed to their large surface-to-volume ratio [3,4,5,6]. Among these materials, magnetic nanoparticles (MNPs) are particularly intriguing due to their oxidase-like, peroxidase-like, catalase-like, and/or superoxide dismutase-like activities, which hold great potential for biological and medical applications [7].

Compared to naturally occurring peroxidase enzymes, enzyme-like nanoparticles exhibit significantly greater stability and maintain an almost unchanged catalytic activity even under harsh conditions [8]. Additionally, peroxidase enzymes are challenging to produce in large quantities. Conversely, inorganic nanoparticles can be easily synthesized in large yields and at a relatively low cost. Given these advantages, nanoparticles have the potential to replace peroxidases in various applications, including those that rely on the detection of hydrogen peroxide and glucose.

However, artificially synthesized nanoparticles, produced through chemical means, have certain drawbacks such as lower purity, easy aggregation, and challenges in coupling with biomolecules. Consequently, there is an urgent need to develop new nanoparticles that possess high dispersity and good biocompatibility in the field of enzyme-like nanoparticles. This has led researchers to explore the use of microorganisms for nanoparticle synthesis. Among the various methods, the bacterial synthesis of nanoparticles stands out due to its advantages of a short cultivation cycle and simple operation, making it a prominent approach in the biosynthesis of metal nanomaterials [9,10,11,12,13].

Bacterial magnetic nanoparticles have garnered more attention than ordinary artificial magnetic nanoparticles due to their biocompatibility, safety, and superparamagnetic properties [14]. These magnetic nanoparticles have a wide range of applications, including protein separation, drug delivery, immunoassays, gene therapy, and immobilization [15]. Notably, magnetic nanoparticles also exhibit peroxidase-like activity. Guo et al. [16] reported that magnetic nanoparticles demonstrated potential peroxidase activity and could eliminate intracellular reactive oxygen species (ROS) in *Magnetospirillum gryphiswaldense* MSR-1. Similarly, Pan et al. [17] extracted biogenic magnetic nanoparticles from the strain *Burkholderia* sp. YN01 and found that these nanoparticles possessed intrinsic peroxidase-like activity, catalyzing the oxidation of the peroxidase substrate TMB in the presence of H_2_O_2_. As a result, magnetic nanoparticles have been successfully used as peroxidase mimics for H_2_O_2_ and glucose detection, as well as pollutant degradation [18]. It is anticipated that these magnetic nanoparticles may find useful applications as detection tools or in pollutant degradation in the fields of biosensors and wastewater treatment.

In our previous study, a strain of magnetic- nanoparticle-producing bacteria, named *Burkholderia cepacia* CG-1, was isolated from Dongchang Lake in Liaocheng, Shandong Province, China. In this study, magnetic nanoparticles were successfully separated using ultrasonication, and their chemical composition, morphology, and magnetic properties were characterized. Importantly, it was demonstrated that these magnetic nanoparticles exhibited peroxidase-like activity. As a result, the MNPs were successfully utilized for H_2_O_2_ and glucose detection as a peroxidase mimic. All the data indicate that the purified magnetic nanoparticles have the potential to be used in biotechnology, medicine, biochemistry detection, and other biological fields.

## 2. Results

### 2.1. Characterization of Magnetic Nanoparticles (MNPs)

**Figure 1:**
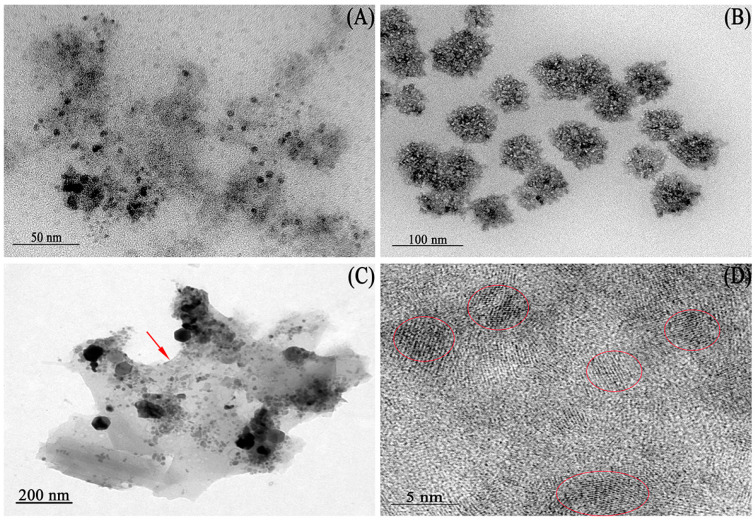
TEM images of the MNPs: (**A**,**B**): The MNPs extracted from the strain *B. cepacia* CG-1 with different incubation times ((**A**) for 3 days and (**B**) for 5 days); (**C**): the membrane structure (the red arrow indicated) surrounding the nanoparticles; (**D**): HRTEM image of the MNPs (the red circles were lattice fringes).

The MNPs were biosynthesized by the strain *B. cepacia* CG-1 (as shown in Figure S1), and their transmission electron microscopy (TEM) images are presented in Figure 1. The images revealed that the sizes of the purified magnetic nanoparticles were uniform, with a distribution of about 4.21 ± 0.09 nm when *B. cepacia* CG-1 was cultured for 3 days (Figure 1A and Figure S2A). However, when the incubation time was increased to 5 days, the bacterial population and vitality increased as the cultivation time increased, resulting in an increase in absorbable Fe ions and an increase in synthesized magnetic nanoparticles [19]. The MNPs formed nanoclusters with an average size of 66.87 ± 0.56 nm (Figure 1B and Figure S2B). Additionally, the TEM image (Figure 1C) showed that the MNPs were surrounded by a membrane (marked with an arrow). This demonstrated that the surface of the nanoparticles had a biofilm; the biofilm activity was strong and attracted other biofilms, leading to the aggregation of nanoparticles into clusters when they existed in the live cell [20]. To further study the microstructure of the MNPs, high-resolution transmission electron microscopy (HRTEM) was used at a magnification of 800,000× and an accelerating voltage of 200 kV. The results revealed that the MNPs were formed by the aggregation of crystals with a size of about 5 nm, and the neat arrangement of atoms could be clearly observed (Figure 1D).

**Figure 2:**
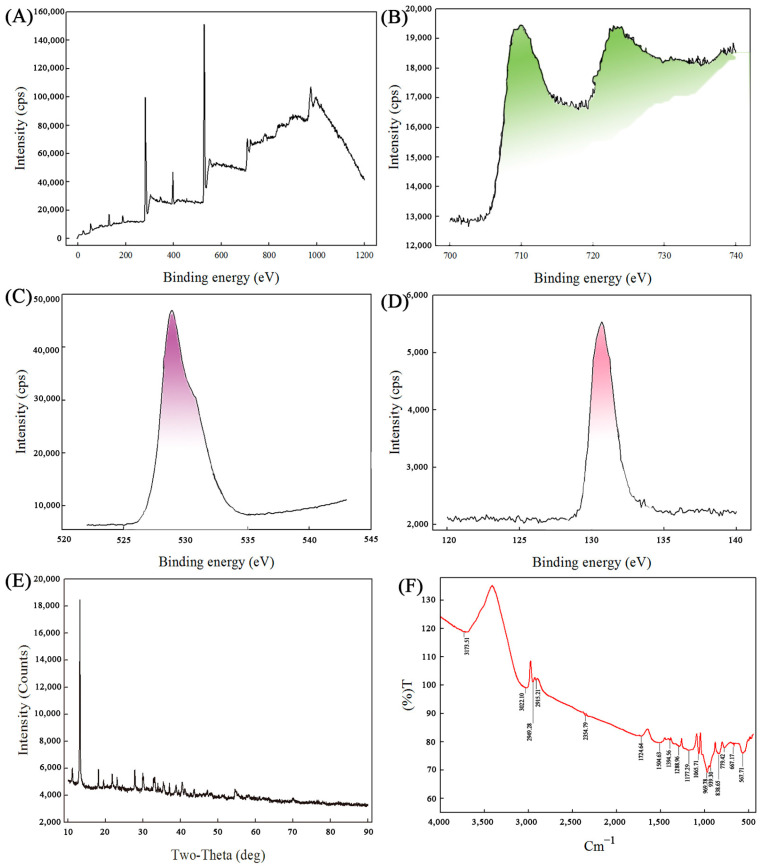
(**A**–**D**): XPS analysis of the MNPs ((**A**): XPS wide spectrum of the MNPs; (**B**): XPS narrow spectrum of Fe 2p; (**C**): XPS narrow spectrum of O 1 s. (**D**): XPS narrow spectrum of P 2p); (**E**): XRD pattern of the MNPs; (**F**): FTIR spectrum of purified the MNPs.

X-ray photoelectron spectroscopy (XPS) analysis was employed to further explore the composition of the MNPs extracted from the strain CG-1. As shown in Figure 2A, there were Fe 2p, O 1s, and P 2p elements present on the surface of the MNPs according to the wide spectrum. The photoelectron peaks at 710 eV and 722.9 eV were identified as the characteristic doublets of Fe 2p3/2 and Fe 2p1/2, respectively, based on the narrow spectrum of Fe 2p (Figure 2B). Additionally, the characteristic peaks at 528.9 eV and 130.7 eV corresponded to O 1s and P 2p, respectively, as shown in Figure 2C and Figure 2D. In addition, there was also a N1S peak present, which may be due to the presence of a small amount of protein during nanoparticle extraction [21].

The structure of the MNPs could be further characterized by XRD. Figure 2E displays the X-ray diffractometer (XRD) patterns of the MNPs sample. The XRD pattern exhibited good crystallinity and closely matched the standard peak for Fe_3_(PO_4_)_2_·8H_2_O (JCPDS 30-0662). Moreover, no significant impurities were detected in the patterns of the MNPs sample, suggesting that the sample was highly purified. Based on these results, the sample was identified as Fe_3_(PO_4_)_2_·8H_2_O.

To gain insights into the surface properties of the MNPs, Fourier transform infrared (FTIR) spectroscopy was utilized to identify the functional groups present on the membrane of the nanoparticles. The results revealed the presence of specific peaks in the wave number range of 500–1500 cm^−1^. These peaks indicated the presence of various functional groups on the surface of the MNPs, including CH_3_ antisymmetric stretching, the NH^4+^ asymmetric variable angle, COO^−^ symmetric stretching, the CH_2_ variable angle of olefin, and PO_3_ symmetry extension (as shown in Figure 2F). These findings were consistent with previous reports that suggested the presence of amino and carboxyl groups on the surface of the nanoparticles. When compared to the characteristic wave numbers of standard Fe_3_(PO_4_)_2_·8H_2_O, there were minor differences in the FTIR spectrum. These differences could be attributed to variations in crystallinity and the presence of other minor components in the sample. Overall, the FTIR analysis provided valuable information on the surface chemistry of the MNPs, which is crucial for understanding their interactions with other materials and their potential applications.

**Figure 3:**
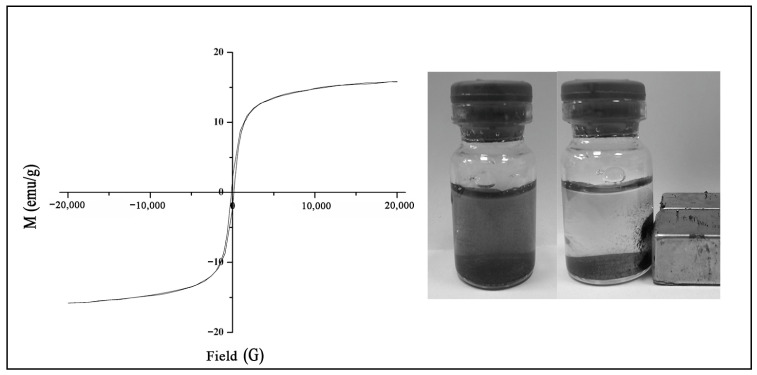
Magnetization curve for magnetic nanoparticles at room temperature.

The hysteresis loop of the MNPs is depicted in Figure 3. The hysteresis parameters, including the coercivity, saturation remanence, and saturation magnetization, were measured to be 151 Oe, 2 emu/g, and 16 emu/g, respectively. These results suggest that the MNPs display ferromagnetic behavior and can be classified as semi-hard magnetic materials. This information is important for understanding the magnetic properties of the nanoparticles and their potential applications in various fields, such as data storage, magnetic separation, and biomedical applications.

### 2.2. Peroxidase-Like Activity of the Fe_3_(PO_4_)_2_·8H_2_O Magnetic Nanoparticles (MNPs)

**Figure 4:**
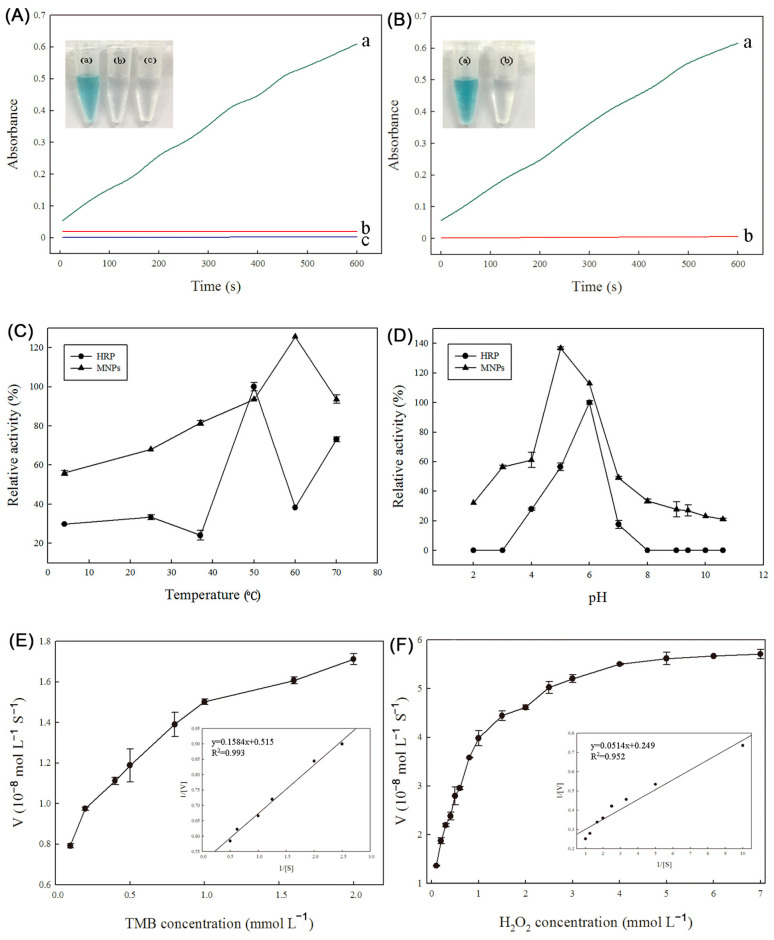
(**A**): The catalytic activities of MNPs in the peroxidase-like oxidation of TMB in NaAc buffer solution (pH 3.8) after 10 min (a: buffer+TMB+H_2_O_2+_Fe_3_(PO_4_)_2_·8H_2_O; b: buffer+TMB+Fe_3_(PO_4_)_2_·8H_2_O; c: buffer+H_2_O_2_+TMB). (**B**): The catalytic activities of MNPs and leaching solution (a: buffer+TMB+H_2_O_2+_Fe_3_(PO_4_)_2_·8H_2_O; b: buffer+TMB+H_2_O_2_+leaching solution). (**C**–**F**): Comparison of the stability of HRP and Fe_3_(PO_4_)_2_·8H_2_O ((**C**): temperature; (**D**): pH). (**E**,**F**): Steady-state kinetic assays of Fe_3_(PO_4_)_2_·8H_2_O. (**E**): The concentration of H_2_O_2_ was constant and the TMB concentration was varied. (**F**): The concentration of TMB was constant and the TMB concentration was varied. Insets are the Lineweaver–Burk plots of the double reciprocal of the Michaelis–Menten equation.

The peroxidase-like activity of the Fe_3_(PO_4_)_2_·8H_2_O MNPs was assessed by observing the catalytic oxidation of the peroxidase substrate TMB in the presence of H_2_O_2_. The characteristic absorption peak for the oxidation of TMB is at 652 nm, which corresponds to a blue color change. As illustrated in Figure 4A, in the absence of either H_2_O_2_ or Fe_3_(PO_4_)_2_·8H_2_O MNPs, the TMB solution remained colorless, indicating that no oxidation reaction took place. However, when the MNPs were added to the reaction mixture containing TMB and H_2_O_2_, a deep-blue color appeared, and the solution exhibited a strong characteristic absorbance at 652 nm. These results demonstrate that the Fe_3_(PO_4_)_2_·8H_2_O MNPs possess intrinsic peroxidase-like activity, as they were able to catalyze the oxidation of the TMB substrate by H_2_O_2_. This finding highlights the potential of these MNPs for applications in biocatalysis, biosensing, and other areas where peroxidase-like activity is required.

To further investigate whether the peroxidase-like catalytic activity of the Fe_3_(PO_4_)_2_·8H_2_O MNPs was due to the intact MNPs or to free metal ions leaching from the particles, an experiment was conducted. The MNPs were incubated in a standard reaction buffer solution with a pH of 3.6 for 10 min. Afterward, the MNPs were removed from the solution using an external magnetic field. The catalytic activity of the resulting leaching solution was then tested, and the result is shown in Figure 4B. The data indicated that there was almost no enzyme activity present in the leaching solution. This finding demonstrates that the intrinsic peroxidase-like activity of the Fe_3_(PO_4_)_2_·8H_2_O MNPs cannot be attributed to the leaching of iron ions into the solution. Instead, the activity occurs on the surface of the MNPs. This result is important for understanding the mechanism of the peroxidase-like activity of the MNPs and confirms that the activity is indeed a property of the intact nanoparticles, rather than a result of free metal ions in the solution. This information can be useful for optimizing the performance of the MNPs in various applications where peroxidase-like activity is required.

### 2.3. Stability of Peroxidase Activity of Fe_3_(PO_4_)_2_·8H_2_O MNPs and HRP

In this study, the stability of the peroxidase activity of the Fe_3_(PO_4_)_2_·8H_2_O MNPs and the natural enzyme horseradish peroxidase (HRP) was evaluated over a wide range of temperatures and pH values. The results revealed that the MNPs exhibited greater stability than HRP. The temperature stability studies showed that the Fe_3_(PO_4_)_2_·8H_2_O MNPs maintained their activity between 37 °C and 70 °C after a 1 h incubation (Figure 4C). On the other hand, the peroxidase activity of HRP decreased significantly when the enzyme was incubated at lower or higher temperatures. Only 30% or 35% of the relative activity remained after HRP was incubated at 25 °C or 60 °C, respectively. Similarly, the Fe_3_(PO_4_)_2_·8H_2_O MNPs were found to be stable over a pH range of 3 to 8 (Figure 4D). However, HRP lost its catalytic activity after being incubated at pH values lower than 3 or higher than 8 for 1 h. These results indicate that, as inorganic nanomaterials, Fe_3_(PO_4_)_2_·8H_2_O MNPs are more stable than the natural enzyme HRP. This stability makes the MNPs a promising candidate for industrial applications where enzymes are required to function under harsh conditions, such as high temperatures or extreme pH values. The potential applications of these stable MNPs in various industrial processes, including biocatalysis, biosensing, and environmental remediation, could be explored further.

### 2.4. Kinetic Assay of Peroxidase-Like Activity of Fe_3_(PO_4_)_2_·8H_2_O MNPs

The peroxidase-like catalytic property of the Fe_3_(PO_4_)_2_·8H_2_O MNPs was further investigated using steady-state kinetics. The kinetic data were obtained by varying the concentration of one substrate and fixing the other substrate concentration. As shown in Figure 4E,F, Michaelis–Menten curves were obtained for Fe_3_(PO_4_)_2_·8H_2_O MNPs with both TMB and H_2_O_2_ as substrates in a certain range of concentrations. As the concentration of TMB or H_2_O_2_ gradually increased, the initial reaction rate *V* gradually increased towards equilibrium, which conformed to the typical Michaelis equation

### 2.5. Reaction Mechanism for the Catalytic Activity of Fe_3_(PO_4_)_2_·8H_2_O MNPs as Peroxidase Mimetics

**Figure 5:**
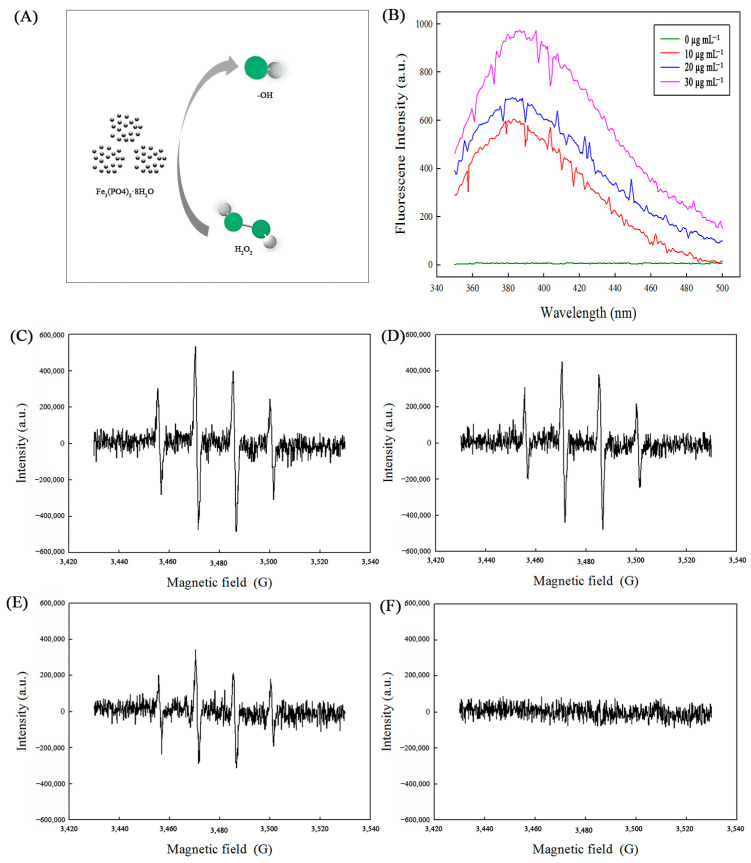
(**A**): Schematic diagram of Fe_3_(PO_4_)_2_·8H_2_O generating ·OH. (**B**): The fluorescence spectra at different concentrations of Fe_3_(PO_4_)_2_·8H_2_O. (**C**–**F**): DMPO spin-trapping ESR spectra of ·OH radicals in the H_2_O_2_-Fe_3_(PO_4_)_2_·8H_2_O reaction system at different concentrations of Fe_3_(PO_4_)_2_·8H_2_O. (**C**): 30 µg mL^−1^ of Fe_3_(PO_4_)_2_·8H_2_O; (**D**): 20 µg mL^−1^ of Fe_3_(PO_4_)_2_·8H_2_O; (**E**): 10 µg mL^−1^ of Fe_3_(PO_4_)_2_·8H_2_O; (**F**): without Fe_3_(PO_4_)_2_·8H_2_O.

To delve into the potential active intermediates within the H_2_O_2_-Fe_3_(PO_4_)_2_·8H_2_O reaction system (Figure 5A), terephthalic acid (TA) was chosen as a fluorescence probe due to its reactivity with ·OH radicals, forming the highly fluorescent 2-hydroxy terephthalic acid (HTA) [22]. Figure 5B illustrates the impact of varying concentrations of Fe_3_(PO_4_)_2_·8H_2_O MNPs on the generation of ·OH radicals, utilizing TA as the fluorescent indicator. Notably, in the absence of H_2_O_2_, no fluorescence intensity was observed. However, upon the introduction of both Fe_3_(PO_4_)_2_·8H_2_O and H_2_O_2_ into the reaction system, a fluorescence became detectable. Furthermore, as the concentration of Fe_3_(PO_4_)_2_·8H_2_O MNPs increased, a corresponding enhancement in the fluorescence intensity was observed. This finding suggests that a greater amount of ·OH radicals was produced in the presence of higher concentrations of Fe_3_(PO_4_)_2_·8H_2_O MNPs, thereby confirming their role in catalyzing the formation of these reactive oxygen species.

Electron spin resonance (ESR) was used to further confirm ·OH radical generation in the H_2_O_2_-Fe_3_(PO_4_)_2_·8H_2_O reaction system (Figure 5C–F). DMPO was used as the spin-trapping agent. The ESR spectra showed a fourfold characteristic peak with an intensity ratio of 1:2:2:1 when the system contained Fe_3_(PO_4_)_2_·8H_2_O MNPs. This specific spectrum was consistent with the pattern of the typical DMPO-·OH adduct [23]. However, no such ESR signal could be observed in control reactions in the absence of Fe_3_(PO_4_)_2_·8H_2_O MNPs. In addition, the ESR signal intensity increased with an increase in the amount of Fe_3_(PO_4_)_2_·8H_2_O MNPs.

Based on the above research, we confirmed that the ·OH radical is the main reactive intermediate in the H_2_O_2_-Fe_3_(PO_4_)_2_·8H_2_O reaction system. Thus, it could be concluded that the peroxidase-like activity of Fe_3_(PO_4_)_2_·8H_2_O actually originates from ·OH radical generation.

### 2.6. Detection of H_2_O_2_ and Glucose Using Fe_3_(PO_4_)_2_·8H_2_O MNPs as Peroxidase Mimetics

Using the intrinsic peroxidase properties of Fe_3_(PO_4_)_2_·8H_2_O MNPs, we designed a simple and sensitive colorimetric method for the determination of H_2_O_2_ and glucose using the Fe_3_(PO_4_)_2_·8H_2_O-catalyzed blue-color reaction. Moreover, this method was used for the detection of glucose in human serum.

**Figure 6:**
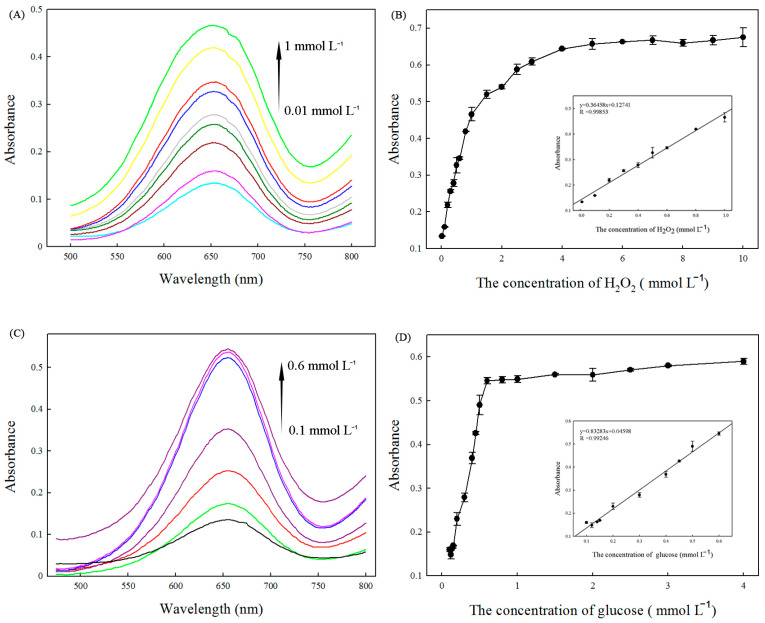
(**A**) The visible spectrum changes in the presence of different concentrations of H_2_O_2_. (**B**): The linear calibration plots for H_2_O_2_ detection. (**C**): The visible spectrum changes in the presence of different concentrations of glucose. (**D**): The linear calibration plots for glucose detection.

Since the catalytic activity of Fe_3_(PO_4_)_2_·8H_2_O is H_2_O_2_-concentration-dependent, this can be used to determine the H_2_O_2_ concentration. As shown in Figure 6A, the absorption intensity of TMB at 652 nm increased as the concentration of H_2_O_2_ varied from 0.01 mmol L^−1^ to 1.0 mmol L^−1^. Figure 6B shows that H_2_O_2_ could be detected at levels as low as 0.001 mmol L^−1^ with a linear range from 0.01 mmol L^−1^ to 1.0 mmol L^−1^. The linear regression equation was A = 0.36458[H_2_O_2_] + 0.12741, and the correlation coefficient R was 0.99853.

The glucose content could be readily detected by utilizing the same chromogenic substrates studied above. In principle, glucose oxidase could catalyze the oxidation of glucose to produce H_2_O_2_. Thus, when the catalytic reaction is coupled with the glucose oxidation reaction by GOx, the change from converted TMB could be used to indirectly measure the glucose content with the aid of Fe_3_(PO_4_)_2_·8H_2_O MNPs as the peroxide-like enzyme. Figure 6C shows the visible spectra of TMB with variations in the concentration of glucose from 0.1 mmol L^−1^ to 0.6 mmol L^−1^. A typical glucose concentration–response curve is shown in Figure 6D. The linear regression equation was A = 0.83283[glucose] + 0.04598 with a correlation coefficient of 0.99246, and the linear range for glucose was from 0.1 mmol L^−1^ to 0.6 mmol L^−1^. The detection limit was 5 × 10^−3^ mmol L^−1^.

**Figure 7:**
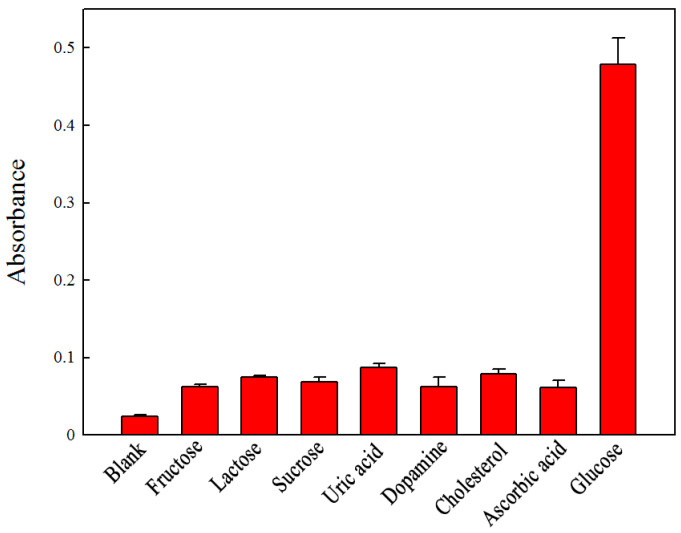
Selectivity analysis of the assay using GOx and the Fe_3_(PO_4_)_2_·8H_2_O MNPs for the detection of glucose. The concentrations of the solutes were 0.5 mmol L^−1^ for glucose and 5 mmol L^−1^ for the other glucose analogues.

To test whether the detection of glucose is specific, control experiments were performed using fructose, lactose, sucrose, lithic acid, dopamine, cholesterol, and ascorbic acid. The selectivity of the colorimetric method is shown in Figure 7. The results showed that there was an obvious color change for the solution containing 0.5 mmol L^−1^ glucose. For the solutions containing 5 mmol L^−1^ control samples, no obvious absorption or color could be observed, which could be attributed to the high affinity of GOx for glucose. These results confirmed that the proposed assay had good selectivity toward glucose.

In an attempt to explore the practical applications of Fe_3_(PO_4_)_2_·8H_2_O MNPs, the Fe_3_(PO_4_)_2_·8H_2_O MNPs-H_2_O_2_ system was applied to determine the glucose level in human blood serum samples. Five serum samples with different concentrations of glucose were diluted 10 times to bring the glucose concentration in the serum samples within the range of the glucose linear regression equation. As shown in Table 1, the results were satisfactory and agreed closely with the clinical data provided by the hospital. Moreover, the relative standard deviation (RSD) varied in the range of 0.61–4.34%, which indicated that the developed method is reliable. All the results indicated that the Fe_3_(PO_4_)_2_·8H_2_O MNPs-H_2_O_2_ system is an effective and reliable technology for the detection of glucose in complicated serum samples.

## 3. Discussion

Magnetic nanoparticles (MNPs) have recently drawn great interest due to their unique features [24]. Some magnetotactic bacteria are believed to produce ferromagnetic nanoparticles via a biomineralization process. In this study, we found that the strain *B. cepacia* CG-1 had high efficiency in producing magnetic nanoparticles inside its cells. However, our earlier study confirmed that another *Burkholderia* sp. YN01, had the ability for magnetic nanoparticle production [17]. These results indicated that *Burkholderia* and its related genera might have the potential for magnetic nanoparticle production when the environment contains iron ions.

The MNPs purified in this study were identified as Fe_3_(PO_4_)_2_·8H_2_O. Fe_3_(PO_4_)_2_·8H_2_O is a kind of widespread iron phosphate found as minerals of the iron(II) salt vivianite. A previous report demonstrated that vivianite formation in the presence of Fe(III)-reducing microorganisms is a typical phenomenon, and some electroactive bacteria such as *Geobacter metallireducens* can synthetize vivianite on their cell surfaces [25,26]. However, there is little information concerning microbes that could synthesize Fe_3_(PO_4_)_2_·8H_2_O inside their cells. To our knowledge, this is the first report that nano-scale Fe_3_(PO_4_)_2_·8H_2_O MNPs could be synthesized by *B. cepacia* CG-1 in its cells. Moreover, it was reported that nonmetal oxyacid anions, such as PO_4_^3−^, have high negative energy, which can bring out a strong inductive effect and thus contribute to electronic conduction and reaction with H_2_O_2_ [27,28]. This phenomenon indicates that Fe_3_(PO_4_)_2_·8H_2_O MNPs has the potential to act as a peroxidase-like enzyme.

The enzyme activity and properties of biosynthesized Fe_3_(PO_4_)_2_·8H_2_O MNPs were studied for the first time in this investigation. It was found to have peroxidase-like activity, similar to that of other iron-based enzymes [5]. The Fe_3_(PO_4_)_2_·8H_2_O MNPs exhibited peroxidase-like activity, catalyzing the oxidation of TMB in the presence of H_2_O_2_. It is well documented that artificial magnetic iron oxide nanoparticles, such as Fe_3_O_4_, can mimic the function of peroxidases, catalyzing the oxidation of various peroxidase substrates like TMB, di-azo-aminobenzene (DAB), and o-phenylenediamine (OPD), resulting in color changes similar to those observed with horseradish peroxidase (HRP) [2]. Like that of other iron-based enzymes, the peroxidase-like activity of Fe_3_(PO_4_)_2_·8H_2_O is concentration-, pH-, and temperature-dependent. Compared to naturally occurring peroxidases, iron oxide nanoparticles offer significantly greater stability across a wide range of pH and temperature conditions. Additionally, their magnetic properties enable easy recovery and recycling. Our earlier research confirmed that Fe_3_O_4_ nanoparticles extracted from the strain *Burkholderia sp.* YN01 could be used as peroxidase mimics [19]. Li et al. [11] reported enhanced peroxidase-like activities of magnetic nanoparticles under visible-light irradiation. However, the mineral cores of the MNPs purified in these studies were composed of Fe_3_O_4_. No previous research has explored whether other types of MNPs, consisting of different iron oxide compounds, possess enzyme-like activities. In this study, we demonstrated for the first time that MNPs composed of Fe_3_(PO_4_)_2_·8H_2_O exhibit intrinsic catalytic activity. This novel type of biogenic MNPs may provide new insights into the formation of MNPs within cells and hold great potential for medical and biotechnological applications.

In our study, both the fluorescence spectroscopy and ESR analysis confirmed that the ·OH radical is the main reactive intermediate in the H_2_O_2_-Fe_3_(PO_4_)_2_·8H_2_O reaction system. Moreover, it is worth mentioning that there was no hydroxyl radical production when the system contained only H_2_O_2_. Based on these observations, we concluded that Fe^2+^ catalyzed the conversion of H_2_O_2_ to ·OH radicals based on the Fenton reaction as follows:

Fe^2+^ + H_2_O_2_ → Fe^3+^ + OH^−^ + ·OH

Then, the generated ·OH oxidized TMB into the typical blue color. This mechanism was not consistent with that of the reported inorganic nanomaterials, originating from electron transfer.

It is widely accepted that many peroxidase mimetics, especially organic polymers and inorganic nanomaterials, could be developed for H_2_O_2_ and glucose detection [29]. Compared to HRP, artificial enzymes usually have much lower detection limits for the substrates. Thus, peroxidase mimetics have received considerable attention in recent years [30]. However, there have been few reports concerning whether biogenic magnetic nanoparticles could be used as biosensors to detect H_2_O_2_ and glucose. In our study, we successfully established a novel platform for the colorimetric detection of these substrates. The results demonstrated that the biosensing system is highly sensitive for H_2_O_2_ and glucose detection. The detection limits were calculated to be 1 μmol L^−1^ for H_2_O_2_ and 5 μmol L^−1^ for glucose, which were lower than those of systems based on Fe_3_O_4_, Por-Ceria, nitrogen-doped graphene, H_2_TCPP-NiO, and Cu (Table 2). Based on this outcome, the bio-synthesized Fe_3_(PO_4_)_2_·8H_2_O can be applied to medical detection in the future.

## 4. Materials and Methods

### 4.1. Chemicals

TMB, horseradish peroxidase (HRP, EC1.11.1.17, 250–330 U mg^−1^), and glucose oxidase (GOx, EC 1.1.3.4. 47, 200 U mg^−1^) were obtained from Sigma-Aldrich (St. Louis, MI, USA). Hydrogen peroxide (H_2_O_2_, 30%) was obtained from Aladdin Regent Company (Shanghai, China). Serum samples were obtained from Northeast Forestry University Hospital. All of the other chemicals used were of analytical grade or the highest quality available. Ultrapure deionized (DI) water was used throughout the experiments.

### 4.2. Bacterial Strain and Culture

The strain that was used in this study was isolated from Dongchang Lake, Liaocheng, Shandong Province, China (36.45° N, 115.97° E). The isolate was identified as *Burkholderia cepacia* by using 16S rDNA sequencing analysis and was designated as *B. cepacia* CG-1.

The medium used for magnetosome production contained the following chemicals: succinic acid 0.74 g L^−1^, NaNO_3_ 0.25 g L^−1^, KH_2_PO_4_ 0.68 g L^−1^, CH_3_COONa 0.12 g L^−1^, Na_2_S_2_O_3_ 0.05 g L^−1^, ferric citrate 12.25 mg L^−1^, Wolfe’s mineral solution 5 mL L^−1^, and Wolfe’s vitamin solution 10 mL L^−1^ [39].

### 4.3. Production and Extraction of Magnetic Nanoparticles

The strain *B. cepacia* CG-1 was inoculated into a 250 mL flask containing 190 mL of magnetic nanoparticle production medium for static incubation at 30 °C. After 5 days, the bacterial cells were harvested by centrifugation at 4 °C (10,000× *g* r/min) and washed twice with 100 mmol L^−1^ phosphate buffer (pH 7.0). The pellet was resuspended in the same buffer. The cells were disrupted by sonication at 4 °C (5s, 60% output, 200×). Magnetic nanoparticles were separated from the disrupted cells with a permanent magnet. After collection, the MNPs were washed with phosphate buffer (pH 7.0) and dried at 60 °C under vacuum for 12 h.

### 4.4. Characterization of Magnetic Nanoparticles

The morphological investigations of the newly exacted MNPs were carried out via transmission electron microscopy (HRTEM, Tecnai G2 F30, Hillsboro, OR, USA) at an accelerating voltage of 200 kV. The X-ray diffraction analysis of the MNPs was performed on an X-ray diffractometer (XRD, Rigaku, D/max-rB, Tokyo, Japan) using Cu K*α* radiation (*λ* = 1.5418 Å). A Fourier transform infrared analysis of the MNPs was carried out via Fourier transform infrared spectroscopy (FTIR; Bruker, Karlsruhe, Germany) using the transmission mode, and the scanning range was 400–4000 cm^−1^. An X-ray photoelectron spectroscopy (XPS) analysis was conducted with an AXIS ULTRA PLD spectrometer (Kratos Co., San Diego, CA, USA) using Al as the exciting source at 1486.4 eV with a pass energy of 10 eV. Polluted carbon was used in charged corrections of the binding energy of the MNP sample at 284.6 eV. Room-temperature magnetic experiments were performed on a vibrating sample magnetometer, and the hysteresis loop was measured at 20,000 Oe. The saturation magnetization (MS), saturation remanence (MrS), and coercive force (HC) were determined after correction for paramagnetic phases. Low-temperature demagnetized experiments were performed on a Quantum Design Magnetic Property Measurement System.

### 4.5. Kinetic Analysis

To further investigate the catalytic kinetics of the Fe_3_(PO_4_)_2_·8H_2_O MNPs-based system, the kinetic parameters of the peroxidase-like reaction were measured by the enzyme kinetics theory and methods. The typical Michaelis–Menten curves were recorded under the same conditions by varying the concentration of one substrate, H_2_O_2_ or TMB, while keeping the other substrate constant. Kinetic constants such as the Michaelis–Menten constant (*K*m) and maximal velocity (*V*max) were calculated from Lineweaver–Burk plots [40].

### 4.6. Comparison of the Stability of Fe_3_(PO_4_)_2_·8H_2_O MNPs and HRP

In this study, we evaluated the stability of Fe_3_(PO_4_)_2_·8H_2_O MNPs and HRP under different pH and temperature values. The pH stability was determined by measuring the activity remaining after the incubation of Fe_3_(PO_4_)_2_·8H_2_O MNPs or HRP for 1 h in buffers of various pH values (2.2–10.6). The thermal stability of Fe_3_(PO_4_)_2_·8H_2_O MNPs and HRP was determined by conducting the assay in 0.1 mol L^−1^ phosphate buffer (pH 7.0) for 1 h at 4–70 °C. After incubation, the enzymes’ remaining activities were measured under their optimized conditions.

### 4.7. Measurement of Hydroxyl Radical Formation

For fluorescence spectroscopy, 20 μL of 1 mmol L^−1^ terephthalic acid, 20 μL of 100 mmol L^−1^ H_2_O_2_, and different concentrations of magnetic nanoparticles were added into 10 mmoL^−1^ NaAc buffer (pH 3.6) and incubated for 15 min at room temperature. After that, the solutions were measured by a fluorescence spectrometer.

For electron spin resonance, 20 μL of 40 mmol L^−1^ 5,5-Dimethyl-1-pyrroline N-oxide (DMPO), 20 μL of 100 mmol L^−1^ H_2_O_2_, and different concentrations of magnetic nanoparticles were added into 10 mmoL^−1^ NaAc buffer (pH 3.6) and incubated for 10 min at room temperature. Samples for ESR spectroscopy were injected into quartz capillary tubes placed in the ESR cavity. DMPO was used to trap the hydroxyl radicals (·OH) to form the DMPO/·OH spin adduct.

### 4.8. H_2_O_2_ Detection Using MNPs as Peroxidase Mimetics

A typical colorimetric detection for H_2_O_2_ was realized as follows: Initially, 20 μL of 1 mg mL^−1^ MNPs, 20 μL of 25 mmol L^−1^ TMB, and 40 μL H_2_O_2_ with different concentrations were added into 530 μL of 200 mmoL^−1^ NaAc buffer (pH 3.6). The mixture was then incubated at 50 °C for 3 min. The resulting solution was used for adsorption spectroscopy measurements at 652 nm.

### 4.9. Glucose Detection Using MNPs and Glucose Oxidase

Glucose detection was performed as follows: Initially, 20 μL GOx (5 mg L^−1^) and 180 μL glucose of different concentrations in 10 mmol L^−1^NaAC buffer (pH 5.5) were incubated at 37 °C for 30 min to produce H_2_O_2_. Afterwards, 20 μL TMB (25 mmol L^−1^), 30 μL MNP stock solution (1 mg mL^−1^), and 350 μL of 200 mmoL^−1^ NaAC buffer (pH 3.6) were added into the above solution. Finally, the mixture was incubated at 55 °C for 30 min and then used for adsorption spectroscopy measurements at 652 nm.

For glucose determinations in serum, the serum samples were firstly treated by centrifugation at 3000× *g* r/min for 20 min. Then, each sample was diluted tenfold using 10 mmol L^−1^ phosphate buffer (pH 7.0) for the following work. According to the above procedure, the glucose in serum was measured.

## Figures and Tables

**Table 1:** Determinations of glucose contents in human serum.

Sample	Provided by Hospital (mmol L^−1^)	Experimental Result (mmol L^−1^ ± SD, *n* = 3)	RSD (%)
1	5.6	5.45 ± 0.04	0.69
2	6.1	6.10 ± 0.08	1.27
3	5.6	5.73 ± 0.25	4.34
4	5.8	5.72 ± 0.04	0.61
5	5.5	5.35 ± 0.11	2.07

**Table 2:** Comparisons of this work with other nanomaterial-based enzyme mimics for the detection of H_2_O_2_ and glucose.

Nanomaterial	Linear Range (μmol L^−1^)	Detection Limit (μmol L^−1^)	Reference
SDS-MoS_2_	2–100 *	0.32 *	[31]
	5–500 **	0.57 **	
Fe_3_O_4_	5–100 *	3 *	[32]
	50–1000 **	30 **	
H_3_PW_12_O_40_	0.134–67 *	0.134 *	[33]
	0.1–100 **	0.1 **	
Por-Ceria	10–100 *	1.8 *	[34]
	40–150 **	19 **	
Nitrogen-doped graphene	20–1170 *	5.3 *	[35]
	25–375 **	16 **	
Graphene oxide	0.05–1 *	0.05 *	[36]
	1–20 **	1 **	
H_2_TCPP-NiO	20–100 *	8 *	[37]
	50–500 **	20 **	
Cu	10–1000 *	10 *	[38]
	100–2000 **	100 **	
Fe_3_(PO_4_)_2_·8H_2_O	10–1000 *	1 *	This work
	100–600 **	5 **	

* For the detection of H_2_O_2_; ** for the detection of glucose.

## Data Availability

The data are available from the corresponding author upon request.

## Supplementary Materials

The following supporting information can be downloaded at: https://www.mdpi.com/article/10.3390/ijms252312518/s1.
