# Supplementary material for: Fast Colorimetric Detection of H2O2 and Glucose: A Way Based on Magnetic Nanoparticles Composed of Fe3(PO4)2·8H2O Isolated from Burkholderia cepacia CG-1
Source: Int J Mol Sci. 2024 Nov 21;25(23):12518. doi: 10.3390/ijms252312518 (PMC11641181; doi:10.3390/ijms252312518)
Supplement: Supplementary file 1 [file ijms-25-12518-s001.zip › ijms-3251002-supplementary.pdf]

# Fast Colorimetric Detection of $H_2O_2$ and Glucose: A Way Based on Magnetic Nanoparticles Composed of $Fe_3(PO_4)_2·8H_2O$ Isolated from *Burkholderia cepacia* CG-1

Mingyu Jia [†], Jueyu Wang [†], Yuxuan Liu, Daizong Cui * and Min Zhao *

College of Life Science, Northeast Forestry University, Harbin 150040, China; 18724626298@163.com (M.J.); 18804503512@163.com (J.W.); liu1959146010@163.com (Y.L.)

* Correspondence: daizongcui@nefu.cn (D.C.); zhaomin@nefu.cn (M.Z.); Tel.: +86-451-82191513 (M.Z.); Fax: +86-451-82191513 (M.Z.)

† These authors contributed equally to this work.

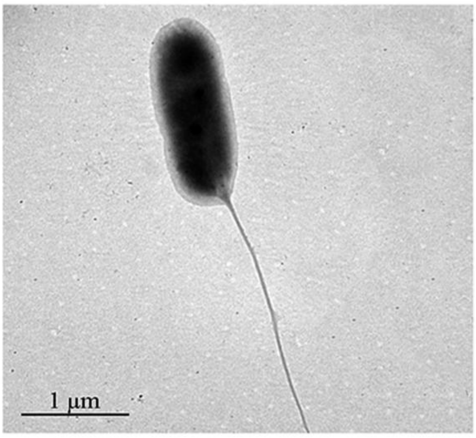


Fig S1 The SEM of *B. cepacia* CG-1

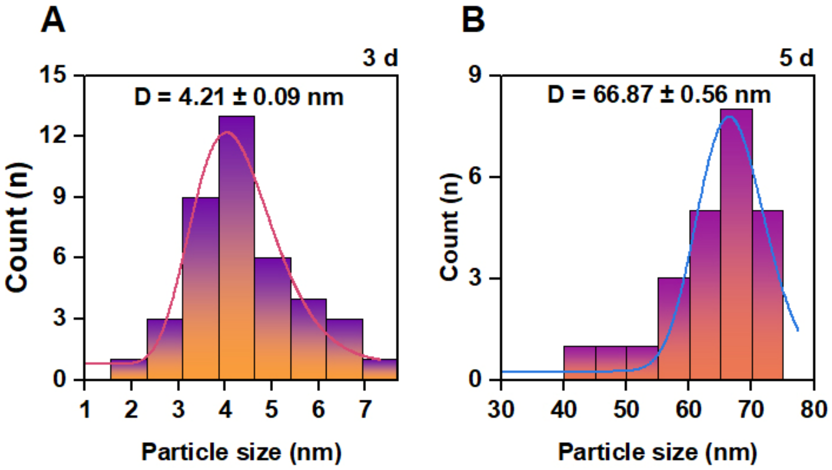


Fig S2 The size of $Fe_3(PO4)_2$ • $8H_2O$ nanoparticles
